# eLIPS: Development and Validation of an Observational Tool for Examining Early Language in Play Settings

**DOI:** 10.3389/fpsyg.2020.01813

**Published:** 2020-07-31

**Authors:** Lynne G. Duncan, Conny Gollek, Douglas D. Potter

**Affiliations:** ^1^Psychology, School of Social Sciences, University of Dundee, Dundee, United Kingdom; ^2^School of Education and Social Sciences, University of the West of Scotland, Paisley, United Kingdom

**Keywords:** language development, child-centered, language difficulties, observation, action research, classroom evaluation, play, early years

## Abstract

Intervention in the early years can help to mitigate the risks that early language and communication difficulties pose for later learning and well-being. Critical to this is the capacity of early years educators to evaluate language development accurately in the classroom in order to target individual support effectively. This article reports on the development and testing of the Early Language in Play Settings (eLIPS) tool, an observational measure of child language. An action research model was used in the design of the tool with the result that the methodology adopted was compatible with an early years child-centered approach. Observations of children in play settings were used to gather information about early language through subscales measuring social communication, receptive and expressive language. A series of preliminary trials with 3- to 5-year-olds, established that the eLIPS measures have concurrent validity with scores from a standardized language assessment, the Clinical Evaluation of Language Fundamentals-Preschool 2^*UK*^. Investigation of internal consistency showed reliability for use by researchers and early years educators together with inter-rater reliability across these groups. It was concluded that eLIPS has potential as a tool to assist early years educators in understanding individual patterns of language acquisition in a play-based environment and for framing team discussions about approaches to early language support.

## Introduction

Creating an effective learning environment for communication and language development is fundamental to achieving quality and equity in early childhood education (Schleicher, 2019). This paper describes a project in which an action research methodology brought together participants from practice and research backgrounds to examine how to raise awareness of language development in a way that would impact practice in the playroom. The outcome is a novel observational tool, *Early Language in Play Settings* (eLIPS), which formalizes the knowledge of early years educators and promotes discussion about the next steps for each child.

The review below summarizes the research background to this project. The value of directing support to language and communication in early education has been established by a large body of longitudinal research, which has documented the relationship between early expressive and receptive language skills and subsequent developmental and societal trajectories (Rescorla, 2011; Yew and O’Kearney, 2013). Further, some children have been identified as being at particular risk for later language difficulty such as those from families with low socio-economic status (SES) (Pace et al., 2017). Early years educators play a key role in supporting language development but, as will be outlined below, the task of identifying those most at risk in the early years is not straightforward and the availability of appropriate tools to guide practice is limited.

### Early Language: A Foundation for Later Development

Clear links have been reported between the language skills that children exhibit in the early years and the subsequent course of language acquisition. Early expressive language as measured by parental report of vocabulary, e.g., MacArthur-Bates Communicative Development Inventories (CDI, Fenson et al., 2007), is one of the strongest predictors of later expressive skills (Rescorla, 2011). Expressive vocabulary has received particular attention in longitudinal cohort studies, where group differences in vocabulary size at age 2 years have been found to persist later in preschool (Dale et al., 2003; Reilly et al., 2010) and at least as far as age 11 (Lee, 2011; Duff et al., 2015). Small vocabularies at age 2 are linked to syntactic and other grammatical weaknesses when measured over short time intervals within preschool as well as over much longer intervals as far as ages 13 and 17 years (Bates and Goodman, 1999; Rescorla, 2005, 2009; Rescorla and Turner, 2015). Other early language skills such as standardized measures of receptive vocabulary at age 4 can predict later performance at age 6 (Dollaghan and Campbell, 2009), and early grammar, when measured by the ability to combine words at age 2, is a predictor of vocabulary and narrative ability outcomes at age 8 (Poll and Miller, 2013).

Weak early language skills have been implicated in lower literacy and math outcomes up to a decade later (Young et al., 2002; Duff et al., 2015). With regard to literacy, typical language development lays a strong foundation for later achievement (National Center for Family Literacy, 2009). Models such as the Simple View of Reading (Gough and Tunmer, 1986) capture this relationship by describing reading comprehension as dependent on both written word identification skill and oral language comprehension. Recent longitudinal evidence for this theory shows that early (receptive and expressive) language at age 4 predicts later written word identification indirectly via letter-sound skills, and language at age 7 predicts reading comprehension, not only concurrently but also its rate of growth between 7 and 9 years (Hjetland et al., 2019). The longitudinal design of such studies enables a causal interpretation with the possibility that this link between language and reading is bidirectional in nature as weak reading skills further compound existing language difficulties (Nation, 2019).

In parallel with attainment outcomes, a literature has emerged charting the consequences of weak early language skills for socio-emotional development. Meta-analyses demonstrate that children with early language difficulties have an elevated risk of clinical levels of emotional, behavioral and attention deficit hyperactivity problems in later childhood and adolescence (Yew and O’Kearney, 2013; Chow et al., 2018). Early expressive skills at age 2 predict concurrent emotional and behavioral difficulties (Whitehouse et al., 2011) and longitudinal prediction can be improved by the addition of receptive language measures (e.g., Clegg et al., 2015). More specifically in social communication development, children whose language includes the use of mental-state terms at age 2$\frac{1}{2}$ are more likely to show understanding of theory of mind at age 4 (Brooks and Meltzoff, 2015). However, children who suffer early language difficulties are at risk of higher levels of externalizing behaviors and later conduct problems, which in turn can constrain their subsequent language development (Menting et al., 2011; Girard et al., 2016; Levickis et al., 2018).

While the mechanisms mediating the links between early language and socio-emotional development are still under investigation, early language problems that constrain interaction may increase the risk of rejection by peers, a factor that has been shown to mediate the link between poor language and later externalizing behaviors (Menting et al., 2011). Further, as early language skills are associated with the emergence of concern for others, including prosocial behavior and empathy (Rhee et al., 2013; Girard et al., 2016; Levickis et al., 2018), poor language may lead to difficulty in perspective taking and emotion recognition which could in turn explain the link to disregard for others (Rhee et al., 2013). Finally, given the role of language in the emergence of self-regulation (emotional and behavioral), weak language skills are likely to lead to difficulties in communicating needs or to delays in the development of the control processes associated with internalizing private speech (Vallotton and Ayoub, 2011; Petersen et al., 2013; Yew and O’Kearney, 2013).

Some children are especially vulnerable to early language difficulties, which, if unsupported, can place them in danger of social isolation and underachievement in later life. Common risk factors are reviewed in the next section.

### Risk Factors in Early Language Development

A family history of Developmental Language Disorder (DLD) places preschool children at significant risk due to the genetic contribution to persistent language difficulties (Bishop, 2006; Bishop et al., 2017). The severe and persistent language difficulties that define DLD significantly interfere with everyday life with no clear biomedical etiology and a poor prognosis (see Bishop et al., 2016, 2017, for a full definition). Since DLD emerges in the course of development, a diagnosis is not usually possible in very young children under the age of 4 years but as children grow older, predictive accuracy improves (Bishop et al., 2017).

While early risk factors are not robust predictors of DLD, they are statistically associated with language difficulties, in that they are more frequent among children with language difficulties than among those developing typically (Bishop et al., 2017). As well as highlighting biological risks (e.g. genetic factors), a cohort study by Christensen et al. (2017) confirmed the influence of clusters of environmental risk on receptive vocabulary development between preschool and the initial years of schooling, especially the well-established impact of financial and material inequalities.

Children who grow up in poverty are particularly vulnerable to early language difficulties and delays (Hart and Risley, 1995; Hoff, 2006). Parental report reveals significant delays in expressive vocabulary among infants from less advantaged families as early as 18 months, which, by the age of 2 years, had grown into a delay of 6 months (Fernald et al., 2013). Standardized tests further corroborate associations between income, vocabulary and grammatical skills (Morisset et al., 1990; Dollaghan et al., 1999). A survey of entrant nursery school children in areas of social and economic deprivation found that over 50% had core language difficulties (CELF-Preschool; Clinical Evaluation of Language Fundamentals, Wiig et al., 1992), which were moderate to severe in scale despite average non-verbal abilities (Locke et al., 2002). The inclusion of SES as a predictor either at an individual or cohort level, strengthens the accuracy of early language measures in predicting later language outcomes (Rescorla, 2011; Heath et al., 2014). Evidence also suggests that the effects of SES may be cumulative, becoming even more important as the child grows older, likely due to increased exposure to disadvantaged environments (e.g., Reilly et al., 2010).

Although considerable variation exists within both high and low socio-economic groups, cohort-level SES differences in language outcome are well-established and thought to be mediated by differences in the quantity and quality of child-directed speech (e.g., Hart and Risley, 1995). Detailed studies of the content of caregiver speech support this view. For example, diversity in the vocabulary and syntactic structures used by caregivers stimulates growth in these aspects of language between 14 and 46 months and the higher the quantity (frequency) of exposure to such elements, the earlier is their emergence in child speech (Huttenlocher et al., 2010). Rowe (2012) provides further developmental detail in showing that, at 18 months, the quantity of parental input is critical for later receptive vocabulary development but, by 30 months, it is the quality of input in terms of vocabulary diversity and sophistication that is most influential. Importantly, in Rowe’s study both of these factors were subject to SES-related differences. Evidence of the impact of caregiver speech fits with a social-constructivist or social-interactionist perspective on language development (Vygotsky, 1978; Bruner, 1983; Hoff, 2006), which proposes that children are sensitive to the linguistic input within their social context and that the features of this language experience determine variation in the course of language development.

### Educational Support for Language in the Early Years

Early years education has a critical role in the provision of support for language development. Low SES children, especially the most disadvantaged, who miss out on this provision or have poor attendance, are likely to have weaker language and later literacy skills (Buckingham et al., 2014; Roy et al., 2014). Variation in the quality of this early years provision also has an observable impact on preschoolers’ language development and school attainment (Buckingham et al., 2014).

As would be predicted from the social-interactionist perspective outlined above, children whose teachers deliver high quality language input make more progress in language learning than children in less supportive teaching environments. Huttenlocher et al. (2002) reported a direct relationship between the amount of complex syntax used by pre-school teachers and the extent of growth in syntactic comprehension among the children in their classrooms. Teachers who use sophisticated vocabulary during free play with 4-year-olds can boost later receptive vocabulary and emergent literacy skills with longitudinal benefits for school reading accuracy and comprehension (Dickinson and Porche, 2011). The use of decontextualized language with preschoolers such as narrative talk about past and future events has also been shown to have benefits for later receptive vocabulary (Rowe, 2012).

By adopting these linguistic techniques as a universal teaching strategy, early years educators can provide a rich environment for the development of language and social communication skills, as well as helping to compensate for the reduced quantity and quality of input experienced by many young children from disadvantaged backgrounds. Nevertheless, as Roy et al. (2014) highlight, the quality of preschool provision can be further enhanced by also training educators “to recognize the presence, nature and significance of language problems and how best to respond and intervene” at the level of the individual child. While being involved in a high-quality language environment is important for every child, the benefits of the higher-level forms of teacher input described above may not be immediately accessible to younger preschoolers or children with language difficulties. The study by Rowe (2012) indicated that the type of caregiver input that was most beneficial was contingent on the child’s age and language level. This is consistent with the idea of adults scaffolding the development of children’s language skills (Vygotsky, 1978; Bruner, 1983) by adjusting their choice and use of language to a level just beyond the child’s current point of development in order to facilitate language growth.

To do this effectively requires building a positive relationship with the child to understand their perspective on the world. A key part of this process is insight into the child’s current level of language acquisition and the significance of this level for educational practice (Brown, 2014). Access to methods for identifying preschool language skills could assist early years educators in aligning practice with learning level when planning classroom and individual interventions. An overview of existing methods is presented in the next section.

### Measuring Language Skills to Guide Practice

As reviewed previously, parental report of early expressive vocabulary using the CDI is one of the strongest predictors of later expressive language (Rescorla, 2011). An extension of this approach asks early years educators to reflect on and report their memory of a child’s vocabulary and decontextualized language use (e.g., future tense) in a qualitatively similar way (CDI-Edu; Bleses et al., 2018). This method is successful but only suitable for children up to the age of 3 years as beyond this point it becomes increasingly difficult to specify all words and language used and understood by a child, whether as a parent or as a teacher.

Informal approaches based on classroom observations and professional experience have typically been used to guide practice in supporting the early language development of 3- to 5-year-olds (Brodie, 2013). A widely used technique is to maintain a learning journal, which contains a record of observations together with examples of activities and comments (e.g., photos, artwork) to reflect a child’s development across the different areas of the early years curriculum. While language development is part of the curriculum that is monitored, this method is not intended to record systematically the growth of specific components (e.g. phonology, semantics, syntax, pragmatics) of a child’s receptive and expressive language.

Reviews of more formal standardized procedures for assessing language development highlight the importance of gathering information across a range of early language skills since component skills can be differentially delayed or impaired (Dockrell, 2001; Conti-Ramsden and Durkin, 2012; Dockrell and Marshall, 2015). This broader perspective can be gained by using more extensive standardized batteries which encompass both receptive and expressive language skills [e.g., Clinical Evaluation of Language Fundamentals-Preschool-2 (CELF-Preschool-2; Wiig et al., 2004); New Reynell Developmental Language Scales (NRDLS; Edwards et al., 2011)]. Areas of concern can be followed up with more specialized assessments, for example: social communication as implicated in Autism Spectrum Disorder (ASD) [e.g., Descriptive Pragmatics Profile, CELF-Preschool-2; Early Sociocognitive Battery (Roy and Chiat, 2019)]; phonological and morphosyntactic processing abilities [Early Repetition Battery (Seeff-Gabriel et al., 2008)]; and vocabulary [e.g., Receptive vocabulary: Peabody Picture Vocabulary Test - Fifth Edition (PPVT-5; Dunn, 2018); and both Receptive and Expressive Vocabulary: Wechsler Preschool and Primary Scale of Intelligence–Fourth Edition (WPPSI–IV; Wechsler, 2012)].

The advantages of standardized tests are well documented (e.g., Kaplan and Saccuzzo, 2009). By following a standard procedure for every child, performance can be compared with age-matched norms to give insight into typical and atypical performance. While clinical diagnosis for DLD or ASD is the responsibility of agencies other than early years educators, early identification for referral to these agencies and for guiding intervention is a core objective of practice. Furthermore, evidence-based practice highlights the need for pre- and post-tests to evaluate intervention efficacy.

While there are advantages to the use of standardized assessments, there are barriers to adopting this approach in early education. One is credentialing – many of the assessments have rules restricting their use to individuals with qualifications which are not customary among early years educators. A notable exception to this is a standardized screening tool designed specifically for educators to use in assessing receptive and expressive language, Wellcomm: Early Years (Hurd and McQueen, 2010). Another barrier is a perceived incompatibility between early education and formal testing, which creates a difficulty or reluctance among early years educators in integrating the use of standardized assessments within their social or play-based pedagogy (Wall et al., 2015).

A more general critique of standardized assessments of early language presents a further barrier, namely, their low predictive power. As an illustration, Dale et al. (2003) followed children who had been identified as language-delayed using the CDI at age 2 and found that only 40% were still classified as delayed at age 4. Dockrell and Marshall (2015) take up this point in their review paper, flagging two criteria central to screening accuracy: (i) sensitivity – the ability to identify children who have language difficulties; and (ii) specificity – ability to avoid misidentifying children as suffering from a language difficulty. Even widely used batteries such as the CELF-Preschool 2 are subject to limitations in predictive accuracy, with sensitivity of only 64% and specificity of 93% between 4 and 5 years (Eadie et al., 2014). In addition to measurement error, individual variability in language development seems likely to be a factor in the low predictive accuracy since children can show early difficulties that subsequently resolve (possibly partially as a result of early educational intervention) or can suffer difficulties that emerge only later in development (Rescorla, 2011).

### The Present Study

A clear trade-off exists for practice between the low prediction rates of persistent language difficulties from standardized assessment in the early years and the need to intervene to support poor language as early as possible due to the risk for later literacy and socio-emotional outcomes. Conti-Ramsden and Durkin (2012) acknowledge that less precise but nevertheless informative guides to language development can be sufficient for non-clinical goals like educational practice. Such a tool would retain the potential to contribute to critically reflective practice by enriching adult-child interactions and discussions with colleagues and partners (e.g., Paige-Smith and Craft, 2011). It could also complement more formal standardized assessment and thereby enhance the interdisciplinary sharing of information with other services.

This study was a partnership with a local education authority to design such a tool, with the following aims:

1 To provide an overview of individual variation in developing language skills among 3- to 5-year-olds.

2 To assist in identifying children who are either already experiencing or at risk of developing language difficulties.

3 To be suitable for use by early years educators as part of their practice, with the potential to raise awareness about the course of language acquisition.

## Phase 1: Development

### Materials and Methods

A collaborative action research model (Kemmis et al., 2014) was adopted for the development phase, which brought university researchers and masters students in psychology together with early years educators, educational psychologists and early education coordinators for a series of eight workshops across a two-year period. A steering group was also formed which met to contribute to the project three times per year and comprised senior education officers and representatives from other agencies (e.g., speech and language therapists, health visitors) as well as from the action research group.

Seven early years settings elected to take part, having been invited to participate due to having sufficient space to accommodate students and researchers, a large enough intake and being representative of the range of socio-economic contexts within the region in Scotland in the United Kingdom. University researchers and students visited these setting regularly to make observations of the children’s language usage and classroom practice, and to trial the materials under development.

In keeping with the action research methodology, a cyclical process was initiated to develop the measure of early language. This adhered to the general methodological structure of planning, acting, observing the outcome of the action and then reflecting on the process before re-planning to begin the cycle again (Kemmis et al., 2014). The first workshops were reflective explorations of theory and practice relevant to the project. Each involved presentations by university researchers or educators followed by small-group discussions around the following topics: (1) perspectives on language development; (2) methods of early years assessment; (3) language use in playroom activities; and (4) classroom working practices. Wherever necessary, questions that emerged were investigated further using observational techniques in the classroom or interviews with early years educators.

### Results

The main themes that emerged from the workshop discussions in relation to the new tool design are outlined below:

#### Child-Centered Classrooms

Early years educators drew attention to the emphasis on child-initiated experiences in their settings. Children are actively encouraged to choose their own play activities according to their interest and motivation. Adults engage with the children during play to scaffold their learning rather than directing classroom activities to deliver instruction.

#### Relevance for Practice

The need for a shared understanding about language development emerged. Early years educators emphasized the importance of having a classroom perspective on language development as the classroom would be integral to both measurement and subsequent practice.

#### Workload

Early years educators placed a strong emphasis on language development but the time available for retraining and tracking this component of the early years curriculum was acknowledged to be limited. Discussion about pre-existing professional skills and monitoring techniques explored efficient solutions.

#### Features of Existing Tools

Observational methods were familiar to the early years educators either because they were used already or else featured in discussions with other services [e.g., Personal Learning Journey (PLJ); Every Child A Talker (Department for Children Schools and Families, 2008); Strengths and Difficulties Questionnaire (Goodman, 2001)]. Tools that require children to be extracted from play in order to engage them in the direct assessment tasks were not perceived as consistent with classroom practice (e.g., CELF Preschool 2; Wellcomm: Early Years), although the depth of information supplied was valued.

### Discussion

The dialogue among action research group members grounded the research within the early years context, establishing key shared priorities. The first concerned child-centered practice, which helped in establishing play as the interactional context for the new tool. This choice is consistent with a social-constructivist theoretical perspective on child development and learning. Theorists such as Vygotsky emphasize the importance of socio-dramatic play for the child, since “play creates a zone of proximal development of the child. In play a child always behaves beyond his average age, above his daily behavior; in play it is as though he were a head taller than himself” (Vygotsky, 1978, p. 102). Empirical support for this view shows that children aged between 4 and 6 years are able to demonstrate more advanced cognitive and socio-emotional skills in play than in non-play situations (for a review see Bodrova and Leong, 2015).

A second priority was to choose methods which are meaningful for practice. Early years educators’ familiarity with categorizing developmental abilities according to benchmarks produced in line with the curriculum offered an opportunity to adapt existing observational methods to early language, thereby minimizing the extra workload. Situating the observations of early language within core playroom activities also seemed likely to promote discussion relevant to understanding each child’s current development and the next steps for that child in educational terms.

A third priority was to examine a range of language skills due to evidence that predictive accuracy of later language difficulty increases when a range of language skills are examined (Rescorla, 2011; Dockrell and Marshall, 2015). Early years educators in the action research group expressed interest in the different linguistic skills assessed by standardized tests, which suggested that observations of these skills in practice might offer an avenue for further increasing awareness about language development.

## Phase 2: Design and Initial Development of the *Early Language in Play Settings* (eLIPS) Measure

The Phase 1 data enabled the design of a series of techniques for measuring language in the playroom, which were subsequently used to gather data and then assessed for potential in meeting the action research group’s priorities.

**FIGURE 1:**
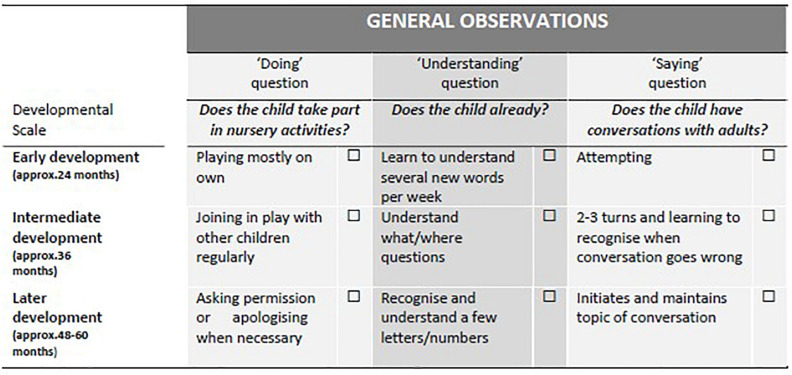
Summary examples of observational questions relating to social communication (Doing), receptive language (Understanding) and expressive language (Saying) for the General Observations play domain.

The goal was for children’s language to be observed while they were playing in core activities of their own choosing. Initially, the House Corner, Sand and Water, and General Observations were identified as suitable play domains, with Outdoors, Snack and Personal Learning Journey (PLJ) added later. For each domain, observational questions were devised for each of three language components: social communication (Doing), receptive language (Understanding) and expressive language (Saying). Each question had a response scale, which reflected theoretical understanding of language development (e.g., Brooks and Kempe, 2012; Rowland, 2013) but in a summarized milestone format more familiar in early years practice and using concrete examples directly related to classroom activities. The task of the observer is to decide where the child would be securely placed on this developmental scale. Although approximate chronological ages were assigned to the scale, as outlined in Figure 1, these would not be visible to the observer.

An iterative process then began of using trial findings and workshop discussions to further refine the tool (see Supplementary Materials for trial details). Early years educators gave feedback about the match between questions and play domains, any ambiguity in wording and the ease of completion. The pilot measure produced is called eLIPS.

## Phase 3: Longitudinal Pilot Study

In the next phase, this pilot version of eLIPS was tested with a larger group of children across one school year. Longitudinal assessment was made of validity and reliability, and specificity and sensitivity data were calculated to determine how well the pilot tool predicted language difficulty.

### Materials and Methods

#### Participants

A total of 78 children (36 girls) from four settings were followed across a school year by trained researchers. Three of the children were bilingual and the rest had English as their first language. Their mean age at Time 1 in September was 41.32 months (*SD* = 2.95, range = 37–49) and at Time 2 in April was 48.41 months (*SD* = 3.01, range = 42–59). Postcode data were collected at Time 1 to assess SES using the Scottish Index of Multiple Deprivation (SIMD), producing a mean quintile score of 2.47 (*SD* = 1.40; range = 1–5). For a subsample of 50 children with attendance data, the SIMD quintile score was found to correlate with attendance during the longitudinal period of the study, *r*(48) = 0.30, *p* = 0.04.

#### Materials and Procedure

The eLIPS Pilot, which comprised six potential play domains (General Observations, House, Outdoors, PLJ, Sand and Water, and Snack) was administered at two time points, 7 months apart. Each child was observed using two of these domains: the General Observations domain for all children plus one other play domain selected according to the child’s own choice of activity in the playroom. The number of questions for Doing, Understanding and Saying in each domain was: General Observations (2, 2,and 2 questions, respectively); House (3, 2, and 3); Outdoors (3, 2, and 2); PLJ (2, 2, and 3); Sand and Water (2, 2, and 3); and Snack (2, 2, and 3). The average number of response choices per question was: General Observations (10); House (9); Outdoors (11); PLJ (11); Sand and Water (9); and Snack (10).

**FIGURE 2:**
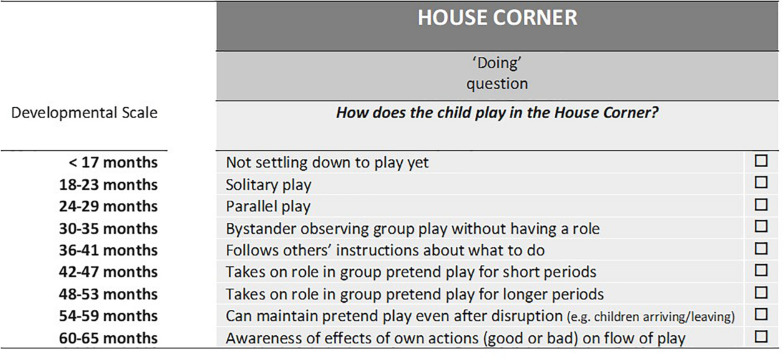
Example of an observational question relating to social communication (Doing) for the House Corner play domain in eLIPS Pilot. For each question, the task is to tick the box describing where the child would be securely placed on the developmental scale.

An example of a Doing question for the House play domain can be inspected in Figure 2. Also shown is the scoring system, which is based on chronological age and arranged in 6-monthly intervals from early to more advanced preschool development. This key is not visible to users when completing the observations. The midpoint of these age bands was used in calculating the eLIPS Pilot age equivalents, by taking the median across questions from the two play domains for each language component to give separate Doing, Understanding and Saying age equivalents, and across all components to produce an overall score, the eLIPS Pilot Early Language age.

The standardized assessment, CELF Preschool 2^*UK*^ (CELF-P2; Wiig et al., 2004), was used as the criterion for assessing concurrent validity. Scaled scores were obtained from three CELF-P2 subtests: Sentence Structure for receptive language; and Word Structure and Expressive Vocabulary for expressive language. These CELF-P2 subtests have been found to be the best discriminators of children with disordered language and together form a Core Language measure (Wiig et al., 2004). The CELF-P2 yields subtest scaled scores with a mean of 10 (*SD* = 3) and a standardized Core language score with a mean of 100 (SD-15). The CELF-P2 Descriptive Pragmatics Profile checklist was also administered for social communication but, as it does not have standardized norms, raw scores were used. To measure non-verbal ability, the British Ability Scales (BAS II; Elliott et al., 1996). Picture Similarities subtest was administered. These tests were conducted at each Time point after completion of the eLIPS Pilot.

### Results

#### Reliability

A reliability analysis was carried out on each eLIPS Pilot play domain (see Table 1). Cronbach’s alpha indicated high internal consistency at both time points for the General Observations, House and Sand and Water domains (all α in range 0.78 to 0.93). The other domains showed more variable results, although each approached an acceptable level of reliability at one time point. It was observed that two of the three less reliable domains had been used with small numbers of children. The items in the less reliable domains (Outdoors, PLJ, Snack), that would increase alpha if dropped, were noted.

**TABLE 1:** Item-total correlations and internal consistency (α) for each of the eLIPS Pilot play domains at Time 1 and Time 2.

**eLIPS domain**		***N***	**Number of items**	**Min.**	**Max.**	**<0.30**	**0.30**–**0.50**	**>0.50**	**Cronbach’s alpha**
General Observations	Time 1	72	6	0.38	0.70	0	1	5	0.80
	Time 2	75		0.41	0.78	0	3	3	0.78
House	Time 1	13	8	0.60	0.89	0	0	8	0.93
	Time 2	18		0.32	0.83	0	1	7	0.89
Outdoors	Time 1	14	7	−0.11	0.58	1	5	1	0.50
	Time 2	15		0.41	0.79	0	1	6	0.86
PLJ	Time 1	9	7	0.08	0.71	3	1	3	0.64
	Time 2	17		0.17	0.79	1	1	5	0.78
Sand and Water	Time 1	17	7	0.65	0.85	0	0	7	0.90
	Time 2	11		0.27	0.83	1	1	5	0.83
Snack	Time 1	13	7	−0.20	0.88	1	2	4	0.74
	Time 2	4		−0.32	0.90	3	3	1	0.40

#### Validity

Time 1 and Time 2 means and standard deviations for the eLIPS Pilot and the CELF-P2 can be inspected in Table 2. The CELF-P2 Core Language measure indicated that the sample were scoring within the low average range at both time points. Early Language age equivalents for the eLIPS Pilot were slightly below actual ages by 1 month at Time 1 and by 4 months at Time 2. The BAS II indicated near average non-verbal T-scores at Time 1 (*M* = 47.09; *SD* = 7.44) and Time 2 (*M* = 49.88; *SD* = 8.64).

**TABLE 2:** Mean group (*N* = 78) age equivalents (months) for the eLIPS Pilot scales (Doing, Understanding, Saying) and Early Language measure and age equivalents (months)^†^ and standardized scores for the CELF-P2 subtests (Descriptive Pragmatics Profile^‡^, Sentence Structure, Word Structure, Expressive Vocabulary) and Core Language measure at Time 1 and Time 2 (standard deviations in parentheses). Concurrent validity coefficients are also shown.

		**Reduced N**		**Time 1**	**Time 2**	**t**	**Pearson r**	
		**Time 1**	**Time 2**	**M (SD)**	**M (SD)**		**Time** **1**	**Time** **2**
**Social Communication**								
**CELF-P2**								
Descriptive Pragmatics Profile (DPP)	*– raw scores*	–	–	64.71 (10.22)	65.08 (10.71)	0.33		
**eLIPS Pilot**								
Doing	*– age equivalent*	–	–	40.08 (6.32)	45.31 (6.90)	6.73***		
eLIPS Doing v. CELF DPP	*– age v. raw score*	–	–				0.53***	0.61***
**Receptive Language**								
**CELF-P2**								
Sentence Structure (SS)	*– scaled score*	–	–	7.56 (3.32)	8.85 (3.05)	3.40**		
	*– age equivalent*	44	63	45.18 (7.77)	49.75 (10.02)			
**eLIPS Pilot**								
Understanding	*– age equivalent (SS reduced sample)*	44	63	44.05 (8.38)	48.24 (6.08)			
	*– age equivalent*	–	–	40.92 (8.73)	46.46 (7.98)	6.66***		
eLIPS Understanding v. CELF SS	*– age v. scaled score*	–	–				0.55***	0.52***
**Expressive Language**								
**CELF-P2**								
Word Structure (WS)	*– scaled score*	–	–	7.72 (3.44)	8.97 (2.90)	4.60***		
	*– age equivalent*	37	63	44.32 (7.93)	48.37 (9.65)			
Expressive Vocabulary (EV)	*– scaled score*	–	–	9.00 (3.64)	9.67 (3.29)	3.20**		
	*– age equivalent*	49	65	45.61 (7.40)	52.89 (10.26)			
**eLIPS Pilot**								
Saying	*– age equivalent (WS reduced sample)*	37	63	39.00 (8.25)	42.24 (8.61)			
	*– age equivalent (EV reduced sample)*	49	65	38.88 (7.40)	42.69 (8.06)			
	*– age equivalent*	–	–	36.31 (8.01)	41.08 (8.80)	5.05***		
eLIPS Saying v. CELF WS	*– age v. scaled score*	–	–				0.30**	0.37**
eLIPS Saying v. CELF EV	*– age v. scaled score*	–	–				0.41***	0.55***
**Overall**								
**CELF-P2**								
Core Language (CL)	*– standard score*	–	–	88.95 (17.39)	94.91 (15.82)	4.78***		
	*– age equivalent^§^*	24	50	47.71 (6.19)	52.07 (8.50)			
**eLIPS Pilot**								
Early Language (EL)	*– age equivalent (CL reduced sample)*	24	50	45.25 (5.15)	47.04 (4.79)			
	*– age equivalent*	–	–	40.15 (6.49)	44.77 (6.59)	6.51***		
eLIPS EL v. CELF CL	*– age v. standard score*	–	–				0.56***	0.62***

*p < 0.05, **p < 0.01, ***p < 0.001. ^†^Age equivalents could not be calculated for children who scored below the 36-month cut-off on the CELF-P2 norms. This accounts for the reduced N at Times 1 and 2. ^‡^There are no standardized scores for the CELF-P2 Descriptive Pragmatics Profile so raw scores are provided. ^§^ There are no age equivalents for the CELF-P2 Core Language measure. The procedure suggested by Wiig et al. (2004) of taking the average of the age equivalents for the three component subtests has been adopted here.

Using independent samples *t*-tests, neither the CELF-P2 Core Language nor the eLIPS Early Language scores showed gender differences or SES differences between high (4 and 5) and low (1 and 2) SIMD quintiles at either time point. Pearson correlations with SIMD quintile scores were significant for CELF-P2 Core Language [Time 1: *r*(76) = 0.27, *p* = 0.02; Time 2: *r*(76) = 0.32, *p* = 0.004] but not for eLIPS Early Language [Time 1: *r*(76) = 0.21, *p* = 0.06; Time 2: *r*(76) = 0.20, *p* = 0.08].

Concurrent validity was tested at each time point using Pearson correlations, which indicated that the eLIPS Pilot had moderate to high concurrent validity with CELF-P2 (see Table 2). The correlation coefficients between all of the eLIPS scores and their comparable CELF-P2 scores were significant but the strongest correlations were found for the Doing and Early Language scores at Time 2.

Age equivalents were calculated for the CELF-P2 Sentence Structure, Word Structure and Expressive Vocabulary subtests, but this reduced the comparison sample size as the CELF-P2 norms do not extend below 36 months. Using this reduced sample, the age equivalents for CELF-P2 and eLIPS Pilot were found to be closest for the receptive language measures (within 1 month at each time point; see Table 2). The comparison was not so close for expressive language, with an average discrepancy of 6 months at Time 1 and 8 months at Time 2 (see Table 2), with eLIPS scores being lower in each case. For comparison with the eLIPS Early Language score, an average age equivalent was calculated from the CELF-P2 subtests to estimate the Core Language age equivalent, a procedure suggested by Wiig et al. (2004). As for all comparisons, the eLIPS ages were below the CELF-P2 ages, with a 2-month and a 5-month gap at Time 1 and 2, respectively.

A repeated measures *t*-test indicated that all of the CELF-P2 and eLIPS Pilot measures, with the exception of the Descriptive Pragmatics Profile, revealed significant growth in language skills across the 7 months of the study (see Table 2).

#### Sensitivity and Specificity

The CELF-P2 Core Language measure was taken as the indicator of language difficulty at each Time point using a cut-off of the standard score being 1 SD or more below the mean (i.e., ≤85). Looking first at the concurrent results in Table 3, the eLIPS Pilot Early Language measure shows higher sensitivity using a 3-month below chronological age cut-off (59%) than a 6-month cut-off (31%), with comparable specificity in each case (>80%). At Time 2, sensitivity is also higher using the 3-month cut-off in eLIPS (89%) but at the expense of specificity (58%); here the 6-month cut-off shows the best outcome with a sensitivity of 68% and a specificity of 83%.

**TABLE 3:** Concurrent sensitivity and specificity calculations for language difficulty at Time 1 and Time 2.

	**Time 1**				**Time 2**			
**(A)**		**CELF-P2 CL ≥ 1SD below mean**				**CELF-P2 CL ≥ 1SD below mean**		
	**eLIPS ≥ 3 months behind CA**	**Yes**	**No**	**Total**	**eLIPS ≥ 3 months behind CA**	**Yes**	**No**	**Total**
	Yes	17	9	26	Yes	17	25	42
	No	12	40	52	No	2	34	36
	Total	29	49	78	Total	19	59	78
	*Sensitivity* = *17/29* = *59%*				*Sensitivity* = *17/19* = *89%*			
	*Specificity* = *40/49* = *82%*				*Specificity* = *34/59* = *58%*			

**(B)**		**CELF-P2 CL ≥ 1SD below mean**				**CELF-P2 CL ≥ 1SD below mean**		
	**eLIPS ≥ 6 months behind CA**	**Yes**	**No**	**Total**	**eLIPS ≥ 6 months behind CA**	**Yes**	**No**	**Total**

	Yes	9	8	17	Yes	13	10	23
	No	20	41	61	No	6	49	55
	Total	29	49	78	Total	19	59	78
	*Sensitivity* = *9/29* = *31%*				*Sensitivity* = *13/19* = *68%*			
	*Specificity* = *41/49* = *84%*				*Specificity* = *49/59* = *83%*			

*At each time point, the number of children identified using a cut-off for the eLIPS Pilot Early Language score of either **(A)** 3 months or more below chronological age or **(B)** 6 months or more below chronological age, is compared with using a cut-off for the CELF-P2 Core Language standard score of 1 SD or more below the mean.*

Longitudinally, the Time 1 CELF-P2 Core Language measure was a very good predictor of language difficulty at Time 2 with sensitivity at 84% and specificity at 78% (Table 4). The results for the Time 1 eLIPS Pilot Early Language scores were lower and best using the 3-month below chronological age cut-off (sensitivity = 58%; specificity = 75%) compared to the 6-month cut-off (sensitivity = 37%; specificity = 83%).

**TABLE 4:** Longitudinal sensitivity and specificity calculations for using Time 1 measures to predict language difficulty at Time 2 as identified by CELF-P2 Core Language standard score of 1 SD or more below the mean.

	**Time 1**	**Time 2**		
		**CELF-P2 CL ≥ 1SD below mean**		
		**Yes**	**No**	**Total**
**(A)**	**CELF-P2 CL ≥ 1SD below mean**			
	Yes	16	13	29
	No	3	46	49
	Total	19	59	78
	*Sensitivity* = *16/19* = *84%*			
	*Specificity* = *46/59* = *78%*			
**(B)**	**eLIPS ≥ 3 months behind CA**			
	Yes	11	15	26
	No	8	44	52
	Total	19	59	78
	*Sensitivity* = *11/19* = *58%*			
	*Specificity* = *44/59* = *75%*			
**(C)**	**eLIPS ≥ 6 months behind CA**			
	Yes	7	10	17
	No	12	49	61
	Total	19	59	78
	*Sensitivity* = *7/19* = *37%*			
	*Specificity* = *49/59* = *83%*			

*The Time 1 predictors were **(A)** the CELF-P2 Core Language score of 1 SD or more below the mean, **(B)** the eLIPS Pilot Early Language score at 3 months or more below chronological age, and **(C)** the eLIPS Pilot Early Language score at 6 months or more below chronological age.*

### Discussion

Moderate to high concurrent validity was found for the eLIPS Pilot at both time points using the CELF-P2 standardized assessment as the criterion. The validity coefficients for all eLIPS measures except for Saying (with CELF-P2 Word Structure) were in the 0.41 to 0.56 range at Time 1 and in the 0.52 to 0.62 range for all measures at Time 2. This longitudinal test of the naturalistic observational methods employed in the eLIPS Pilot has indicated that it performed very well in comparison with an established and more formal standardized assessment. In particular, very good evidence emerged that individual eLIPS Pilot scales were measuring specific components that underpin language development, with the best evidence emerging for receptive language and social communication.

Sensitivity and specificity were calculated using the Celf-P2 Core Language measure as the indication of language difficulty at each time point. For concurrent prediction at Time 1, eLIPS Pilot sensitivity was better when the threshold for identifying language difficulty was 3 months rather than 6 months (59% vs. 31%, respectively), and specificity was very good in each case (>80%). At Time 2, the 3-month cut-off produced very high sensitivity but at the expense of lowered specificity, whereas the 6-month cut-off produced higher levels of both sensitivity (68%) and specificity (83%).

For longitudinal prediction of Time 2 language difficulty, the CELF-P2 Core Language measure proved to be the best Time 1 predictor with very good levels of both sensitivity and specificity (∼80%). The eLIPS Pilot Early Language measure had similar levels of specificity with the best sensitivity evident using the 3-month below chronological age threshold (58%). Although lower than the levels expected for a standardized diagnostic test, the eLIPS Pilot sensitivity values indicate that it is a promising tool for guiding classroom practice.

The eLIPS Pilot and CELF-P2 mean age equivalents were very close for receptive language and for overall scores at Time 1 but less so for expressive language and overall scores at Time 2. Nevertheless, all but one of the eLIPS Pilot measures were within 6 months of the standardized results, which is a good outcome given the nature of the eLIPS Pilot measurement scale. The internal consistency of the eLIPS Pilot was high at both time points for the General Observations, House, and Sand and Water domains (all α in range 0.78 to 0.93). The other domains showed more variable results, which indicated that they require further refinement.

As well as the promising outcomes, the longitudinal study and feedback from early years educators drew attention to clear points to be addressed in the fine-tuning of the eLIPS measure. The expressive language scales, in particular, required modification in view of the lower concurrent validity results. Feedback indicated that questions asking for detailed information about words that children knew proved difficult to complete. As well as further improvements to the wording of items, feedback also revealed a consensus that the PLJ domain had not proved as suitable for playroom use as the other domains and the decision was taken to focus instead on the remaining domains. Finally, items which had produced low internal consistency were reassessed, especially for the Outdoors and Snack domains.

## Phase 4: Reliability and Validity Testing of eLIPS

The final version of eLIPS respects the child-centered nature of the playroom. It consists of four parallel forms to enable educators to respond to the play activity that the child is engaged in. In these parallel forms, the General Observations domain is used together with one other domain selected according to the child’s choice of activity: House, Outdoors, Sand and Water, or Snack.

Data gathered by a trained researcher are presented here to examine the internal consistency and concurrent validity of this final version of eLIPS. A pilot training program was also devised to support early years educators in using eLIPS in the playroom to observe language development. This was a 2$\frac{1}{2}$-hour session run by the trained researchers, which involved watching and discussing video-clips of child language in play settings together with practice and feedback in using eLIPS to record their own observations of social communication, receptive and expressive language. Feedback indicated that the content and format of the eLIPS training session was rated as ‘very good’ on 5-point Likert scales (very poor, poor, okay, good, very good) by 79–87% of the early years educators. The eLIPS data gathered by the trained educators are presented to evaluate internal consistency and inter-rater reliability.

### Internal Consistency and Concurrent Validity for Trained Researcher Use of eLIPS

#### Materials and Methods

##### Participants

A sample of 63 children was recruited from four different settings. A trained researcher conducted eLIPS and then completed the Descriptive Pragmatics Profile for all of the children. Afterward, the CELF-P2 Sentence Structure, Word Structure and Expressive Vocabulary subtests were administered to all but three of the children, who were absent. The final sample consisted of 60 children (23 girls) with complete data. Their mean age was 50.60 months (*SD* = 10.03; range = 37–65).

##### Materials and procedure

The format of the scales was now similar for each domain with two questions each for Doing, Understanding and Saying in each of the General Observations, House, Outdoors, Sand and Water, and Snack domains. Thus, there were six questions in total for each domain. The observational questions retained their original design features (see Figure 2), containing 8–11 points on a developmental scale arranged in order from early to more advanced preschool development. For each child, the researcher would complete two domains: General Observations plus one other domain depending on the child’s choice of activity in the play setting (House, Outdoors, Sand and Water, or Snack). These were the four parallel forms of eLIPS.

A new method of calculating the scores for eLIPS was also tested as the method used in the development and pilot phases proved too cumbersome for playroom use. The goal was for early years educators to be able to have immediate feedback about language skills. Therefore, scores were summed across questions and domains to produce totals for Doing, Understanding and Saying rather than calculating medians as previously had been done by the researchers. The eLIPS Early Language score was calculated as the mean of these three subcomponents and all scores were converted to an age equivalent by means of a reference table based on the developmental scale used in the questions.

This new version was compared with the standardized language assessment, CELF Preschool 2^*UK*^ (CELF-P2) for concurrent validity using the methods described in Phase 3.

#### Results

##### Internal consistency

Internal consistency was assessed separately for each of the parallel forms of eLIPS, all of which used the General Observations domain together with one other play domain: House, Outdoors, Sand and Water, or Snack (see Table 5).

**TABLE 5:** Item-total correlations and internal consistency (α) for eLIPS data gathered by a trained researcher.

**Parallel Form**	**Scale**	**N**	**Min.**	**Max.**	**<0.30**	**0.30**–**0.50**	**>0.50**	**Cronbach’s Alpha**
**House**	**Early Language**	**17**	**0.72**	**0.94**	**0**	**0**	**12**	**0.97**
	Doing		0.77	0.88	0	0	4	0.92
	Understanding		0.88	0.93	0	0	4	0.95
	Saying		0.87	0.96	0	0	4	0.97
**Outdoors**	**Early Language**	**12**	**0.67**	**0.96**	**0**	**0**	**12**	**0.97**
	Doing		0.75	0.90	0	0	4	0.93
	Understanding		0.72	0.93	0	0	4	0.90
	Saying		0.88	0.95	0	0	4	0.96
**Sand and Water**	**Early Language**	**19**	**0.74**	**0.94**	**0**	**0**	**12**	**0.98**
	Doing		0.68	0.92	0	0	4	0.92
	Understanding		0.83	0.97	0	0	4	0.95
	Saying		0.90	0.95	0	0	4	0.97
**Snack**	**Early Language**	**12**	**0.71**	**0.97**	**0**	**0**	**12**	**0.98**
	Doing		0.77	0.95	0	0	4	0.95
	Understanding		0.90	0.95	0	0	4	0.96
	Saying		0.80	0.94	0	0	4	0.95

*Information is tabulated for each of the eLIPS scales (Doing, Understanding, and Saying) and the Early Language measure separately for each of the parallel forms of eLIPS (as determined by the play domain used in conjunction with General Observations).*

##### Validity

Means and standard deviations for eLIPS and the CELF-P2 can be inspected in Table 6. The CELF-P2 Core Language measure indicated that the sample were scoring within the low average range. The mean eLIPS Early Language age equivalent for the full sample was only slightly below actual age (<1 month).

**TABLE 6:** Mean group (*N* = 60) age equivalents (months) for eLIPS scales (Doing, Understanding, and Saying) and Early Language measure and age equivalents (months) and standardized scores for CELF-P2 subtests (Descriptive Pragmatics Profile^†^, Sentence Structure, Word Structure, Expressive Vocabulary) and Core Language measure (standard deviations in parentheses). Concurrent validity coefficients are also shown.

		**Reduced *N***	**Mean (SD)**	**Pearson *r***
**Social Communication**				
**CELF-P2**				
Descriptive Pragmatics Profile (DPP)	*– raw scores*	–	75.72 (10.88)	
**eLIPS**				
Doing	*– age equivalent*	–	50.60 (7.32)	
eLIPS Doing v. CELF DPP	*– age v. raw score*	–		0.64***
**Receptive Language**				
**CELF-P2**				
Sentence Structure (SS)	*– scaled score*	–	7.58 (2.70)	
	*– age equivalent*	48	48.54 (9.95)	
**eLIPS**				
Understanding	*– age equivalent (SS reduced sample)*	48	51.88 (7.83)	
	*– age equivalent*	–	49.20 (9.46)	
eLIPS Understanding v. CELF SS	*– age v. scaled score*	–		0.50***
**Expressive Language**				
**CELF-P2**				
Word Structure (WS)	*– scaled score*	–	8.10 (3.01)	
	*– age equivalent*	41	48.78 (12.83)	
Expressive Vocabulary (EV)	*– scaled score*	–	10.07 (2.89)	
	*– age equivalent*	53	52.77 (13.93)	
**eLIPS**				
Saying	*– age equivalent (WS reduced sample)*	41	53.34 (8.04)	
	*– age equivalent (EV reduced sample)*	53	50.77 (8.72)	
	*– age equivalent*	–	48.70 (10.60)	
eLIPS Saying v. CELF WS	*– age v. scaled score*	–		0.43**
eLIPS Saying v. CELF EV	*– age v. scaled score*	–		0.40**
**Overall**				
**CELF-P2**				
Core Language (CL)	*– standard score*	–	91.58 (13.98)	
	*– age equivalent^‡^*	41	51.42 (10.24)	
**eLIPS**				
Early Language (EL)	*– age equivalent (CL reduced sample)*	41	53.49 (6.43)	
	*– age equivalent*	–	49.40 (8.62)	
eLIPS EL v. CELF CL	*– age v. standard score*	–		0.46***

*p < 0.05, **p < 0.01, ***p < 0.001. ^†^There are no standard scores for the CELF-P2 Descriptive Pragmatics Profile so raw scores are provided. ^‡^There are no age equivalents for the CELF-P2 Core Language measure. The procedure suggested by Wiig et al. (2004) of taking the average of the age equivalents for the three component subtests has been adopted here.

The Pearson concurrent validity coefficients for eLIPS using CELF-P2 as the criterion were all significant and within the moderate to high range (see Table 6). The eLIPS Doing scale showed the highest agreement with the comparable scores from the CELF-P2 assessment (0.70), followed by the Understanding (0.50) and Saying scales (0.40, 0.43). The e-LIPS Early Language score also showed significant concurrent validity with the CELF-P2 Core Language measure at a moderate level of correlation (0.46).

A threshold of 3 months below chronological age again proved the best solution in using eLIPS to identify early language difficulties when 1 SD below the CELF-P2 Core Language standard score mean was used as the criterion. Although specificity was higher using the 6-month threshold, this was at the expense of sensitivity and the balance of both measures (approximately 75%) was much better with the 3-month threshold (see Table 7).

**TABLE 7:** Sensitivity and specificity calculations using **(A)** the eLIPS Early Language score at 3 months or more below chronological age and **(B)** the eLIPS Early Language score at 6 months or more below chronological age to predict language difficulty as identified by CELF-P2 Core Language standard score of 1 SD or more below the mean.

		**CELF-P2 CL ≥ 1SD below mean**		
		**Yes**	**No**	**Total**
**(A)**	**eLIPS ≥ 3 months behind CA**			
	Yes	13	11	24
	No	4	32	36
	Total	17	43	60
	*Sensitivity* = *13/17* = *76%*			
	*Specificity* = *32/43* = *74%*			
**(B)**	**eLIPS ≥ 6 months behind CA**			
	Yes	8	7	15
	No	9	36	44
	Total	17	43	60
	*Sensitivity* = *8/17* = *47%*			
	*Specificity* = *36/43* = *84%*			

### Internal Consistency and Inter-Rater Reliability for Trained Early Years Educator Use of eLIPS

#### Materials and Methods

##### Participants

After participating in the training session, educators from two settings were invited to use eLIPS to collect data from children in their care. Completed forms were returned for 46 children (14 girls) by 18 educators, all of whom were qualified practitioners with at least a Higher National Certificate (HNC) or equivalent. The mean age of the observed children was 41.98 months (*SD* = 4.96, range = 37 – 55).

A trained researcher also collected eLIPS data concurrently from a subsample of this group, which involved 6 educators and 14 children (4 girls; mean age = 38.86 months; *SD* = 1.51, range = 37 – 42).

##### Materials and procedure

When in the play settings, the administration instructions were that eLIPS should be used with children who the early years educator knew well. To achieve this, educators were advised to wait 6 weeks after a child’s entry to the setting before conducting eLIPS. Then observations should be taken afresh so that current rather than remembered information could guide the observations. For each child, the early years educator and the researcher would complete the same two domains: General Observations plus one other domain depending on the child’s choice of activity in the play setting.

#### Results

##### Internal consistency

When implemented by trained early years educators, the parallel forms of eLIPS had excellent internal consistency with α ranging between 0.87 to 0.96 (see Table 8). The scales (Doing, Understanding, Saying) produced similar results, particularly for the House and Outdoors parallel forms. There were three exceptions to this, the understanding and saying scales for Sand and Water and the Doing scale for Snack where internal consistency fell below this level (α in the range 0.56 to 0.68). However, these latter two forms of eLIPS were also administered to the smallest samples.

**TABLE 8:** Item-total correlations and internal consistency (α) for eLIPS data gathered by trained early years educators.

**Parallel form**	**Measure**	***N***	**Min.**	**Max.**	**<0.30**	**0.30**–**0.50**	**>0.50**	**Cronbach’s alpha**
**House**	**Early Language**	**10**	**0.55**	**0.94**	**0**	**0**	**12**	**0.96**
	Doing		0.62	0.73	0	0	4	0.83
	Understanding		0.69	0.91	0	0	4	0.90
	Saying		0.77	0.92	0	0	4	0.93
**Outdoors**	**Early Language**	**22**	**0.65**	**0.90**	**0**	**0**	**12**	**0.95**
	Doing		0.72	0.81	0	0	4	0.97
	Understanding		0.76	0.91	0	0	4	0.93
	Saying		0.71	0.85	0	0	4	0.89
**Sand and Water**	**Early Language**	**6**	−**0.05**	**0.95**	**2**	**2**	**8**	**0.87**
	Doing		0.62	0.92	0	0	4	0.85
	Understanding		−0.14	0.63	1	0	3	0.57
	Saying		−0.09	0.78	2	0	2	0.56
**Snack**	**Early Language**	**8**	**0.23**	**0.87**	**1**	**0**	**11**	**0.92**
	Doing		0.26	0.64	1	0	3	0.68
	Understanding		0.16	0.81	1	0	3	0.76
	Saying		0.58	0.86	0	0	4	0.81

*Information is tabulated for each of the eLIPS scales (Doing, Understanding, Saying) and the Early Language measure separately for each of the parallel forms of eLIPS (as determined by the play domain used in conjunction with General Observations).*

##### Inter-rater reliability

The results contained in Table 9 show a moderate level of agreement between raters for the Doing scale and good agreement for the Understanding and Saying scales. The Early Language measure showed a moderate level of inter-rater agreement.

**TABLE 9:** Intraclass correlation coefficients (ICC) to assess inter-rater reliability for each of the eLIPS scales (Doing, Understanding, Saying) and the Early Language measure (*n* = 14).

	**eLIPS**			
	**Doing**	**Understanding**	**Saying**	**Early Language**
**ICC**	0.42^†^	0.71**	0.63**	0.59*

^†^*p* = 0.06, *p < 0.05, **p < 0.01.

#### Discussion

Results indicated that the parallel forms which comprised this final version of eLIPS had high reliability whether observed by a trained researcher or early years educator (all α in range 0.87 to 0.98). In the researcher-administered study, all three scales for each of the parallel forms had similarly high levels of reliability. This was also true for the House and Outdoors forms in the early years educator study but the reliabilities were lower for Doing (α = 0.68) in the Snack form and for Understanding (α = 0.57) and Saying (α = 0.56) in the Sand and Water form (although participant numbers were also lower for these forms). Inter-rater reliability was found to be moderate to good.

Concurrent validity assessed in the researcher study showed that eLIPS had moderate to high concurrent validity with CELF-P2 (see Table 6). The correlation coefficients between all of the eLIPS scores and their comparable CELF-P2 scores were significant but the strongest correlations were found for the Doing (social communication) and Understanding (receptive language) scores. As in the longitudinal study, the Saying (expressive language) score showed the lowest concurrent validity but was nevertheless significant and moderate in size.

Finally, very good levels of concurrent sensitivity and specificity to language problems (∼75%) were found by using 3-months below chronological age in the eLIPS Early Language measure as the cut-off. While a longer 6-month cut-off increased specificity, the resulting levels of sensitivity were unacceptably low (47%).

## General Discussion

The aim of the present paper was to describe the design, development and preliminary validation of the eLIPS tool, an observational measure of preschool language development. The action research approach was novel, involving early years educators and other potential users of the tool from the earliest phases of the design process. The successive phases of development outlined here suggest that the observational methodology adopted in eLIPS can provide valid and reliable information about language acquisition across social communication, receptive and expressive language, in a format that is adapted to a child-centered playroom.

### Insight Into Individual Variation in Language Skills Among 3- to 5-Year-Olds

This research addresses a gap identified in the literature review regarding the small number of tools available to early years educators for following language development among 3- to 5-year-olds. Educators acquire informal monitoring strategies from the observational skills learned during their training and from their later professional experience in the playroom. However, more formal tools such as standardized assessments tend not to be consistent with child-centered practice in the playroom or else are often restricted in focus in terms of age group or language skills assessed (for reviews, see: Conti-Ramsden and Durkin, 2012; Dockrell and Marshall, 2015). eLIPS occupies a space between these informal and formal approaches to provide early years educators with information about the development of three subcomponents of language: social communication, receptive and expressive language.

The innovative use of an action research model in the design process means that eLIPS aligns very well with the daily practice of early years educators. Observation of play is a familiar and highly practiced skill, which is used by educators in supporting child development during the early years. The focus on play also has a strong theoretical underpinning due to the emphasis on the importance of play as a context for learning among this age group (e.g., Vygotsky, 1978). While the play context already has a long-standing role in the assessment of socio-emotional development (Salcuni et al., 2017), it has been relatively neglected in relation to monitoring language development despite recognition of play as an important setting for communication and social interaction (Lillard, 2015). Further, meta-analytic evidence shows robust concurrent and longitudinal associations between both receptive and expressive language and symbolic play during early childhood (Quinn et al., 2018). Therefore, the present study provides educators with a novel tool which capitalizes on their embeddedness in the playroom to examine and support early language development.

This preliminary validation of the tool produced a strong set of results. Validity and reliability information guided decisions throughout the development of the eLIPS tool. The final version of eLIPS had excellent internal consistency (>0.85) when used by either researchers or early years educators. Comparison with the Core Language measure and associated subtests from the CELF Preschool 2^*UK*^ (Wiig et al., 2004), provided a standardized criterion for concurrent validity in the researcher-administered study. Moderate to high validity was found across the component and overall measures of language produced using eLIPS.

### Identifying Children at Risk of Language Difficulties

Despite the general consensus that accuracy in predicting later language difficulty from early language profiles is poor even when using standardized tests, there is widespread agreement on the need to intervene early to support children with language difficulties (Conti-Ramsden and Durkin, 2012; Dockrell and Marshall, 2015; ICAN/RCSLT, 2018). Our objective in the present work was to develop a composite language tool to guide professional practice within the early years playroom around this issue. The concurrent predictive accuracy of eLIPS in identifying children at risk of language difficulty was estimated against the CELF Preschool 2^*UK*^, a widely used indicator of language difficulty. The use of 6-months below chronological age as the threshold for concern in eLIPS was rejected due to obtaining higher specificity only at the expense of lower sensitivity. By adopting a 3-month threshold in eLIPS, sensitivity and specificity were both found to be approximately 75%. These concurrent prediction levels are an encouraging outcome for eLIPS. In longitudinal predictions across 7 months of the school year, the 3-month threshold in eLIPS produced a longitudinal sensitivity of 58% and a specificity of 75%. As would be expected, the comparable figures for the standardized CELF-P2 assessment were higher at 84% and 78% respectively. Plante and Vance (1994) suggest that scores of 80–89% are fair and >90% are good.

The primary utility of eLIPS is not likely to be the longer-term prediction of difficulty but rather to provide early years educators with information of sufficient accuracy to assist them in scaffolding the language learning of individual children. eLIPS performs close to the acceptable standards for concurrent sensitivity and specificity. Therefore, as well as offering a focus for reflective practice, it provides additional evidence in identifying those at risk for developmental difficulties and can inform discussions with colleagues and other services about the necessity of and best format for intervention strategies. In other words, eLIPS should further enhance early years educators’ participation in the type of multi-method, multi-informant approach advocated by Conti-Ramsden and Durkin (2012). Greater inclusion of educators’ expertise may help to produce a fuller and more meaningful diagnostic picture, which could be particularly important for low SES children (Conti-Ramsden and Durkin, 2012). For example, early years educators tend to be familiar with the local dialect used by children and their families, and so are in an excellent position to accommodate accurately to the children’s speech variety when making eLIPS observations. This contrasts with more centralized services and more formal psychometric tools (Dockrell and Marshall, 2015).

### Understanding Early Language Development in Relation to Practice

The action research model which informed the development of eLIPS allowed the objective of playroom use by early years educators to be built into the design process. By selecting naturalistic observational methods as the basis of eLIPS, it proved possible to be consistent with child-centered practice, to formalize the existing skills and experience of early years educators and, as a result, to reduce the workload implications of using eLIPS.

Our initial investigations of educators’ use of eLIPS revealed good comparability with the reliability results produced by trained researchers with high levels of internal consistency for each of the parallel forms of eLIPS (α ≥ 0.87). There were also moderate to good levels of inter-rater reliability between educators and researchers in using eLIPS after one formal training session. This outcome is promising but will need to be confirmed in larger-scale trials.

As outlined above, eLIPS is intended as a tool for guiding practice rather than as a means of diagnosing developmental language disorders. Feedback from the action research group and from the headteachers in the settings which trialed educator use of the tool established that eLIPS is useful for promoting discussions among staff about the next steps in supporting a child. Tools providing an overview of different aspects of language are thought to be more useful for practice than language tools which focus on only one area of language (Dockrell, 2001; Conti-Ramsden and Durkin, 2012). Therefore, eLIPS has the potential to contribute to reflective practice by informing developmentally appropriate interactions with individual children, by fueling consultation between colleagues in the playroom and by helping to create a shared understanding and approach in collaborations across services.

Discussion within the action research group led to a recommendation to use a traffic light system in the future as a guide for practice rather than eLIPS age equivalents. For example, 3–6 months below chronological age would be shown as amber, more than 6-months below chronological age as red but otherwise the outcome would be green. The intention was to retain a pedagogical focus on the next steps for all children rather than on the extent of any individual ‘delay’ or ‘advancement’ for age. This was also felt to be consistent with the literature on the variation in language acquisition and to acknowledge the documented imprecision in predicting later language difficulties among children in this age group across measurement tools (e.g., Rescorla, 2011). Nevertheless, the possibility of using eLIPS age equivalents remains for more quantitative modeling at a cohort level or for the evaluation of interventions, if required.

Alongside the importance of the reliability and validity of any measure used to assess early language, stands the issue of being able to interpret outcomes in an informed manner (e.g., Dockrell, 2001). This capability needs to be built into training and professional development for early years educators who use tools such as eLIPS. When used effectively, tools that provide insight into language use in the playroom can enhance the quality of those early years settings by increasing the precision of language input from educators, improving the scaffolding of early learning and enabling the voice of the child to be heard more clearly.

### Limitations and Future Developments

Despite the promising results from this preliminary validation of the eLIPS tool, a number of areas would benefit from further research and development. Although it had been the intention to include observations of vocabulary in eLIPS, this did not prove possible in the version produced here. This is perhaps not unexpected given that well-established assessments based on this method such as the CDI have only been validated for ages up to 3 years. Our experiments with observations of play-themed vocabularies met with negative feedback from early years educators who did not feel comfortable using this aspect of the tool with the 3- to 5-year-old age group. This omission is unfortunate as we know that vocabulary is a marker of social disadvantage (e.g., Fernald et al., 2013) and one of the strongest predictors of later expressive language, grammar and literacy outcomes (e.g., Lee, 2011). Future research will continue to examine opportunities to integrate vocabulary into the tool.

Our investigation explored the use of an action research model for the development of a tool for use by early years educators in observing language development. While these initial results suggest that eLIPS has considerable potential for use in guiding practice in the early years playroom, the generalizability of the current findings is limited by the sample size. There is clearly a need to improve statistical power by extending the evidence base to explore the tool’s effectiveness over a larger sample and in a variety of different play settings. This would establish the replicability of the present findings and allow the scales to be refined further to increase the psychometric strength of eLIPS. As concerns have been raised about the reliability, validity and sensitivity of the standardized CELF Preschool 2 assessment itself, in spite of the international use of this Early Language measure (Eadie et al., 2014; Dockrell and Marshall, 2015), it might be that future work should assess validity using more than one standardized measure as criteria.

In future work, the goal will be to refine the scales further and to create a digital version to ease the process of recording, collating and interpreting observations^1^. One priority as eLIPS develops will be to increase the inclusiveness of the tool with adaptations to suit the needs and abilities of a more diverse population. It is hoped that there will also be opportunities to learn from education authorities as to how eLIPS can be implemented and integrated with practice to become part of a decision-making process about the next steps in supporting language development.

## Conclusion

Action research was used to design a tool called eLIPS for educator use in following early language development. The preliminary evaluation presented here has shown that eLIPS has excellent reliability and moderate to good validity for use in examining social communication, receptive and expressive language among children aged between 3 and 5 years of age. Internal consistency in initial analyses of educator usage in the playroom is also excellent. Moreover, feedback from educators supports an ecological fit with the child-centered early years environment due to eLIPS being based on observations of children playing at activities of their own choice. Therefore, eLIPS has the potential to empower early years educators to gather information that can guide their own practice and their interactions with other services in supporting the language needs of individual children in their care.

## Data Availability Statement

The raw data supporting the conclusions of this article will be made available by the authors, without undue reservation.

## Ethics Statement

The consent procedures and study protocol were developed in consultation with the Council responsible for the participating settings and approved by the University of Dundee Research Ethics Committee (UREC 13154). Informed consent was sought from the parents of all children who participated using an opt-out procedure. Verbal and non-verbal cues were taken into consideration in obtaining consent from participating children. As well as using information sheets, extra steps were implemented to keep parents informed and engaged throughout the project via direct practitioner communication and regular updates in the form of posters about progress within the early years settings.

## Author Contributions

LD drafted a first version of the current manuscript. All authors contributed to the manuscript revision and approved the final version for submission.

## Conflict of Interest

The authors declare that the research was conducted in the absence of any commercial or financial relationships that could be construed as a potential conflict of interest.

## Footnotes

1 www.eLIPS.org.uk
