# Supplementary material for: eLIPS: Development and Validation of an Observational Tool for Examining Early Language in Play Settings
Source: Front Psychol. 2020 Jul 31;11:1813. doi: 10.3389/fpsyg.2020.01813 (PMC7412886; doi:10.3389/fpsyg.2020.01813)
Supplement: Supplementary file 1 [file Table_1.docx]

Supplementary Material

# Overview of development trials

The focus of Trial 1 was whether the combination of General Observations plus one other play domain (House or Sand & Water) of the child’s own choice could be a good combination for the final format of the new measure. Trial 2 examined three additional play domains identified by the action research group as key interactional contexts in the playroom (Outdoors, Snack, Personal Learning Journey (PLJ)).

These two new versions were trialed by a trained researcher in separate studies. The standardized assessment, CELF Preschool 2^UK^ (CELF-P2; Wiig et al., 2004), was used as the criterion for concurrent validity. After observations were made with the new measure, scaled or raw scores were obtained from three CELF-P2 subtests: Sentence Structure for receptive language; Word Structure and Expressive Vocabulary for expressive language and the Descriptive Pragmatics Profile checklist for social communication.

# Trial 1: General Observations plus one other play domain

## Materials and Methods

The sample consisted of 45 children (20 girls) from one setting with a mean age of 52.64 months (*SD*=7.97, range = 38–64). Each child was observed using General Observations and either the House (N=23) or Sand & Water (N=22) domain. Each of the play domains had Doing, Understanding and Saying questions, although the number of these varied (House (3, 2 and 4 questions, respectively); Sand & Water (3, 3 and 4); General Observations (2, 2 and 4). The average number of response choices per question was: House (9); Sand & Water (8); and General Observations (10). Ages or age bands were assigned to these response choices. The ages or the age-band midpoint was used in calculating the age equivalent scores, by taking the median across questions from the two play domains for each language component to give separate Doing, Understanding and Saying age equivalents, and across all components to produce an overall score, the Early Language age.

## Results

The age equivalents were: Understanding (*M* = 45.80 months, *SD*=10.12) and Saying (*M* = 50.20 months, *SD*=10.44). These correlated well with the scaled scores from the CELF-P2: receptive language (Sentence Structure (*M* = 6.91, *SD*=2.55), *r*(43)= .71, *p*<.001) and expressive language (Word Structure (*M* = 7.56, *SD*=2.86), *r*(43)= .59, *p*<.001; Expressive Vocabulary (*M* = 9.36, *SD*=2.50), *r*(43)= .57, *p*<.001). There was also a strong association between the Doing age equivalent (*M* = 46.87 months, *SD*=8.75) and the CELF-P2 Descriptive Pragmatics Profile raw scores (*M*=71.73, *SD*=10.01; *r*(43) = .78, *p* < .001). These correlations indicated good concurrent validity for each of the three subscales (Doing, Understanding, Saying).

The mean Early Language age was 48.47 months (*SD*=9.70), which had a strong correlation with the CELF-P2 Core Language standard score (*M*= 87.64, *SD*=13.06; *r*(43) = .73, *p* < .001), indicating concurrent validity. The correlations for the subgroups who did the combination of General Observations plus either the House or Sand & Water play domain were *r*(21) = .67, *p* = .001 and *r*(20) = .78, *p* < .001, respectively. Importantly, these correlations were not found to differ significantly using Fisher’s r-to-z transformation, *z*=.73, *p*=.47.

# Trial 2: New play domains

## Materials and Methods

A sample of 35 children (15 girls) from one setting participated with a mean age of 50.20 months (*SD*=6.19; range = 40–62). Thirteen of the children were observed using all three of the new play domains, and the other 22 children were observed using two domains (Outdoors and PLJ: N=10; Snack and Outdoors: N=5; and Snack and PLJ: N=7). Each of these domains had Doing, Understanding and Saying questions: Outdoors (3, 3 and 3 questions, respectively); Snack (3, 2 and 3); and PLJ (3, 3 and 3). The average number of response choices per question was: Outdoors (10); Snack (9); and PLJ (10). The method of scoring was outlined in the previous section.

## Results

The age equivalents were: Understanding (*M* = 42.77 months, *SD*=9.44) and Saying (*M* = 44.14 months, *SD*=10.61). These correlated well with the CELF-P2 scaled scores: receptive language (Sentence Structure (*M* = 6.71, *SD*=2.42), *r*(33)= .63, *p*<.001) and expressive language (Word Structure (*M* = 6.97, *SD*=3.36), *r*(33)= .68, *p*<.001; Expressive Vocabulary (*M* = 8.49, *SD*=3.33), *r*(33)= .67, *p*<.001). There was also a strong association between the Doing age equivalent (*M* = 45.34 months, *SD*=7.83) and the CELF-P2 Descriptive Pragmatics Profile raw scores (*M*=72.69, *SD*=9.34; *r*(33) = .72, *p* < .001). These correlations indicated good concurrent validity for each of the three subscales (Doing, Understanding, Saying).

The Early Language age (*M*= 45.00, *SD*=8.61) showed strong concurrent validity using the standard scores for the CELF-P2 Core Language measure (*M*=84.80, *SD*=16.10, *r*(33)= .72, *p* < .001. The correlations for the subgroups who did 2 or 3 play domains were, *r*(20) = .56, *p* = .007 and *r*(11) = .89, *p* < .001, respectively. The correlation for the 3-domain subgroup was found to be higher using Fisher’s r-to-z transformation, *z*=2.02, *p*=.04.

# Summary

Decisions were taken to use two rather than three of these domains as this appears to provide a parsimonious method of collecting data about early language. While three domains might have provided greater precision, it proved difficult and time-consuming in Trial 2 to gather this amount of data given that the play domains were determined by the child’s own choice of activity. Fewer children were able to be observed using three scales (n=13) than two scales (n=22) during the time available. A further modification in moving to a two-domain version was to use the General Observations domain for all children with one additional activity specific to the individual child’s interest. The resulting parallel versions of the tool that used either the House Corner or Sand & Water as the additional activity appeared to produce comparable outcomes (Trial 1).

Evaluation of the concurrent validity of these trial versions of the tool using the CELF-P2 as our criterion produced outcomes for social communication, receptive and expressive language as well as the Early Language score in the moderate to high range.
